# The safety and efficiency of benzoyl peroxide for reducing *Cutibacterium acnes* in the shoulder: An updated systematic review and meta-analysis

**DOI:** 10.3389/fsurg.2023.1015490

**Published:** 2023-03-10

**Authors:** DingYuan Fan, Jia Ma, XiaoHua Liu, Sheng Zhang, Jin Sun, Yan Li, Bo Jiang, Lei Zhang

**Affiliations:** ^1^ Department of Joint Surgery and Sports Medicine, Wangjing Hospital, China Academy of Chinese Medical Sciences, Beijing, China; ^2^ Beijing University of Chinese Medicine, Beijing, China

**Keywords:** *P. acnes*, *C. acnes*, benzoyl peroxide, clindamycin, shoulder, infection, surgery, chlorhexidine

## Abstract

**Background:**

*Cutibacterium acnes* (*C. acnes*), a common pathogen, contributes significantly to infections in shoulder surgery. Prevention of shoulder infection is crucial to improve postoperative functional recovery and reduce costs. This study aimed to perform a systematic review and meta-analysis to assess the safety and efficacy of 5% benzoyl peroxide (BPO) application in the shoulder to decrease *C. acnes*.

**Methods:**

Three electronic databases were searched as follows: PubMed, Embase, and the Cochrane Library databases. Data extraction for this study was performed by two independent reviewers, and only level I and level II studies were included. The outcome data sources of individual studies were pooled. The fixed-effect model was used to determine the meta-analysis.

**Results:**

There were five level I studies and five level II studies. The results showed that the 5% BPO group had a lower risk of *C. acnes* positivity [OR, 0.21 (0.15, 0.30), *I*^2^ = 24, *p* < 0.00001]. The pooled analysis results showed that there was no significant difference in the ability of 5% BPO and 5% BPO + clindamycin to reduce *C. acnes*. However, the lower rate of adverse events was significantly in favour of the non-BPO group compared with the 5% BPO group.

**Conclusion:**

BPO can decrease *C. acnes* in the shoulder to prevent infection. However, the combination of BPO and clindamycin does not enhance this effect further.

**Level of evidence:**

II, Systematic review and meta-analysis.

## 1. Introduction

Infection following shoulder surgery remains a devastating complication undesired by both surgeons and patients (1, 2). Common risk factors include diabetes, male sex, age under 75 years, previous shoulder arthroplasty, and rotator cuff arthropathy (3, 4). The incidence of shoulder joint infection was approximately 0.9%–1.8%, 3%–4%, and 0.01%–0.3% for primary arthroplasty, revision arthroplasty, and shoulder arthroscopy, respectively (5–10). Prosthetic infections (PJIs) are often more challenging to manage than postarthroscopic infections. Once infection events occur, poor functional outcomes, disability, more extended hospital stays, and higher costs after surgery are inevitable (11–13). The diagnostic process of infection is complex and time-consuming, involving the integration of clinical symptoms, laboratory exams, radiological studies, and microbiological swabs (4).

Currently, the organism responsible for microorganisms in PJI is *Staphylococcus aureus*, *Staphylococcus epidermidis*, coagulase-negative *Staphylococci*, and *Cutibacterium acnes* (*C. acnes*) (4, 14). *C. acnes* is the most implicated pathogen in shoulder PJI (14–17). The discovery and treatment of *C. acnes* are complex compared to that of other organisms easily diagnosed in PJI and treated with two-stage revisions (14). In addition, these bacteria inhabit the pilosebaceous units of the normal skin, and this part is also a challenging area for shoulder preoperative skin sterilization (18–20). Therefore, reducing the number of *C. acnes* organisms before shoulder joint arthroplasty is essential to preventing PJI.

Benzoyl peroxide (BPO) consists of white crystal agglomerates that are soluble in chloroform or in other organic solvents such as organic peroxides (21). BPO was first identified in the 19th century and is now recommended by dermatologists to treat *C. acne* (22).

Previous studies on the inclusion of BPO were limited, and the evidence level was low, which lacked convincing evidence (23, 24). As several new studies (25–28) have been published, an updated systematic review and meta-analysis should be conducted. This study aimed to assess the safety and efficiency of BPO application in the shoulder to decrease *C. acnes*. The primary outcome was the rate of positivity for *C. acnes*, and the secondary outcome was complications. We hypothesized that BPO would significantly decrease *C. acnes* in the shoulder.

## 2. Methods

### 2.1. Identification and selection of trials

Two independent investigators performed the literature search based on the 2020 Preferred Reporting Items for Systematic Reviews and Meta-Analysis (PRISMA) guidelines, and the PRISMA checklist was used (29). The third investigator resolved any discrepancies. A comprehensive search was performed in three databases, Embase, PubMed, and the Cochrane Library, until the last check on July 1st, 2022 (PROSPERO: CRD42021261880). The following search terms were used {[(Benzoyl Peroxide) OR (Adapalene)] OR (Benzoyl Peroxide Drug Combination)} AND (Shoulder). The titles, abstracts, and full texts were screened, and the reference lists of all included studies were also checked to ensure that no studies were missed.

### 2.2. Inclusion criteria

We included studies that met the following criteria:

**P**: Participants ≥18 years;
**I**: Any treatment that contains 5% BPO;
**C**: No-treatment shoulder, or shoulder receiving other treatment without 5% BPO or with placebo, or shoulder before treatment;
**O**: Rate of positivity for *C. acnes*, adverse events (including any abnormal signs and symptoms);
**S**: Level I and II evidence studies.

### 2.3. Exclusion criteria

(1) Review studies;
(2) Animal studies;
(3) Cadaver studies;
(4) Biomechanical studies;
(5) Cohort studies;
(6) Case report or case series studies;
(7) Full text unavailable;
(8) Not published in English;
(9) Conference abstracts;
(10) Clinical registration records.

### 2.4. Data extraction

Two blinded investigators independently extracted the characteristics of the included studies. The third investigator resolved any disagreement. The following information was recorded: first author's name, year, journal, age, gender, LOE (level of evidence), and BPO intervention. Statistical analyses of this study were performed using RevMan 5.3 software (Cochrane Collaboration) for data management.

### 2.5. Risk of bias

We followed the Cochrane Handbook for Systematic Reviews of Interventions, and the Cochrane risk-of-bias tool was used for all included studies (30). This tool categorized bias into six domains, and each domain was assigned a level of risk of bias (low risk, unclear risk, and high risk). The Kappa score was used to calculate the degree of agreement between reviewers (31). A score of 0–0.20 represents poor agreement; 0.21–0.40, fair agreement; 0.41–0.60, moderate agreement; 0.61–0.80, good agreement; and 0.81–1.00, perfect agreement.

### 2.6. Data synthesis and analysis

This systematic review's results were prioritized using a fixed-effects model; dichotomous data were calculated as odds ratios (OR) with a 95% confidence interval (CI). If there was more than one non-BPO control group in a study, we only extracted data from one control group and preferentially selected the placebo group for analysis. The *I*^2^ statistic was used to quantify heterogeneity: 0%–40% low heterogeneity, 40%–60% moderate heterogeneity, and >60% high heterogeneity. All these values can be examined *via* forest plots.

#### 2.6.1. Subgroup analysis

To better explain the effects of BPO, we classified the different uses of BPO as follows:

1. 5% BPO vs. non-BPO Non-BPO group including a control group, the condition before use employed as the comparison group, a chlorhexidine gluconate group, and a pHisoHex group (1% triclosan; sodium benzoate, 5 mg/ml; and benzyl alcohol, 5 mg/ml)
2. 5% BPO + clindamycin vs. non-BPO
3. 5% BPO + blue Light vs. non-BPO
4. 5% BPO + chlorhexidine/alcohol vs. 2% chlorhexidine/alcohol.

#### 2.6.2. Publication bias

To examine the possibility of publication bias, a funnel plot was used.

## 3. Results

**Figure 1:**
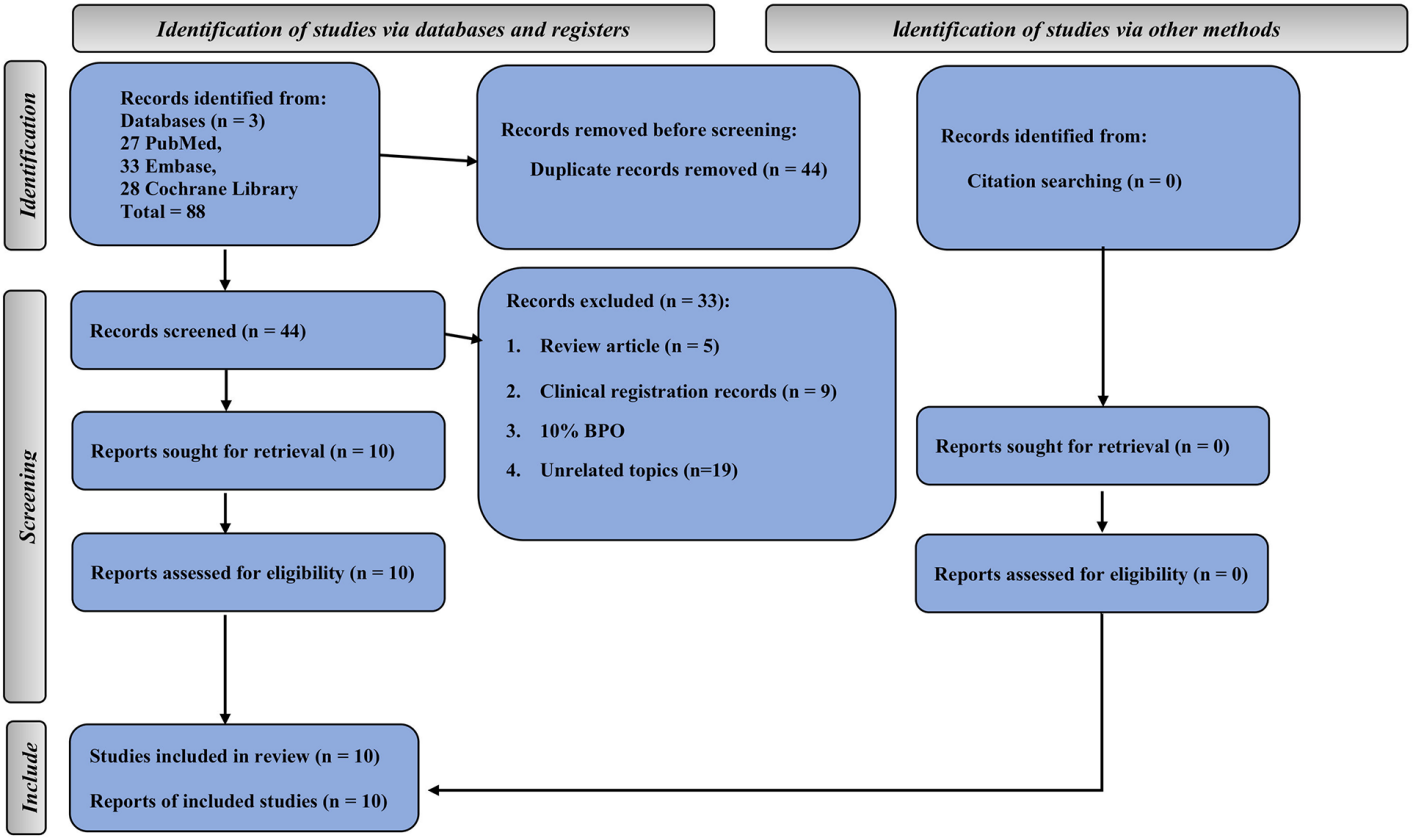
2020 preferred reporting items for systematic reviews and meta-analyses flow chart.

The comprehensive search yielded 88 studies from three databases (27 from PubMed, 33 from Embase, and 28 from the Cochrane Library). Half of these were duplicate studies. In addition, five studies were review articles, 9 studies were clinical registration records, 1 study used 10% BPO, and 19 studies had unrelated topics. Finally, 10 studies met our inclusion criteria (25–28, 32–37) (Figure 1).

### 3.1. Study characteristics

There were five level I studies (26, 27, 33, 35, 37) and five level II studies (25, 28, 32, 34, 36). A total of 9 studies were from the same journal. Two studies included only males (26, 32). Only one paper reported that BPO was used between 1 and 10 times (Table 1).

**Table 1:** Study characteristics.

First author's name	Year	Journal	Age	Total number of total patients	Gender	BPO intervention	LOE
Dizay HH et al.	2017	Journal of Shoulder and Elbow Surgery	BPO group 56.9 (28–79)^a^	65	43 M 22 F	5% BPO + Clindamycin (1–10 times in 1–10 days)	II
Scheer et al.	2018	Journal of Shoulder and Elbow Surgery	20–66^b^	40	24 M 16 F	5% BPO (5 times in 3 days)	I
Hancock DS et al.	2018	ANZ Journal of Surgery	30^c^	22	22 M	5% BPO + Chlorhexidine/Alcohol (1 time in 1 day)	II
Kolakowski L et al.	2018	Journal of Shoulder and Elbow Surgery	BPO group 51 (23–77)^a^ CHG group 51 (18–88)^a^	80	BPO group 18 M 23 F CHG group 19 M 20 F	5% BPO (3 times in 3 days)	I
Heckmann et al.	2019	Journal of Shoulder and Elbow Surgery	29.4 (26–40)^a^	12	10 M 2 F	5% BPO (6 times in 3 days) 5% BPO + Clindamycin (6 times in 3 days)	II
van Diek FM et al.	2020	Journal of Shoulder and Elbow Surgery	BPO group 55.1 ± 9.1^d^ Placebo group 56 ± 6.8^d^	30	BPO group 5 M 10 F Placebo group 6 M 9 F	5% BPO (5 times in 3 days)	I
Scheer et al.	2021	Journal of Shoulder and Elbow Surgery	BPO group 63 ± 13^d^ Control group 65 ± 13^d^	100	BPO group 23 M 22 F Control group 40 M 15 F	5% BPO (5 times in 3 days)	II
Cotter EJ et al.	2021	Journal of Shoulder and Elbow Surgery	BPO group 26.8 ± 3.62^d^ BLT group 26.7 ± 2.99^d^ BPO + BLT group 25.90 ± 2.97^d^	60	60 M	5% BPO (5 times in 3 days) 5% BPO + BLT (5 times in 3 days)	I
Symonds T et al.	2022	Journal of Shoulder and Elbow Surgery	BPO group 70 ± 5.9^d^ BPO + clindamycin group 67.9 ± 7.6^d^ pHisoHex group 68.1 ± 6.9^d^	99	BPO group 22 M 11 F BPO + clindamycin group 24 M 11 F pHisoHex group 15 M 16 F	5% BPO (5 times in 2.5 days) 5% BPO + Clindamycin (5 times in 2.5 days)	II
Unterfrauner I et al.	2022	Journal of Shoulder and Elbow Surgery	BPO group 61.26 ± 19.70^d^ Control group 56.93 ± 23.28^d^	60	BPO group 12 M 18 F Control group 15 M 15 F	5% BPO (7 times in 7 days)	I

BLT, blue light therapy; BPO, benzoyl peroxide; CHG, chlorhexidine gluconate; F, female; LOE, level of evidence; M, male; NR, not report.

^a^ Mean (range).

^b^ Range.

^c^ Mean.

^d^ Mean ± standard deviation.

### 3.2. Risk of bias

**Figure 2:**
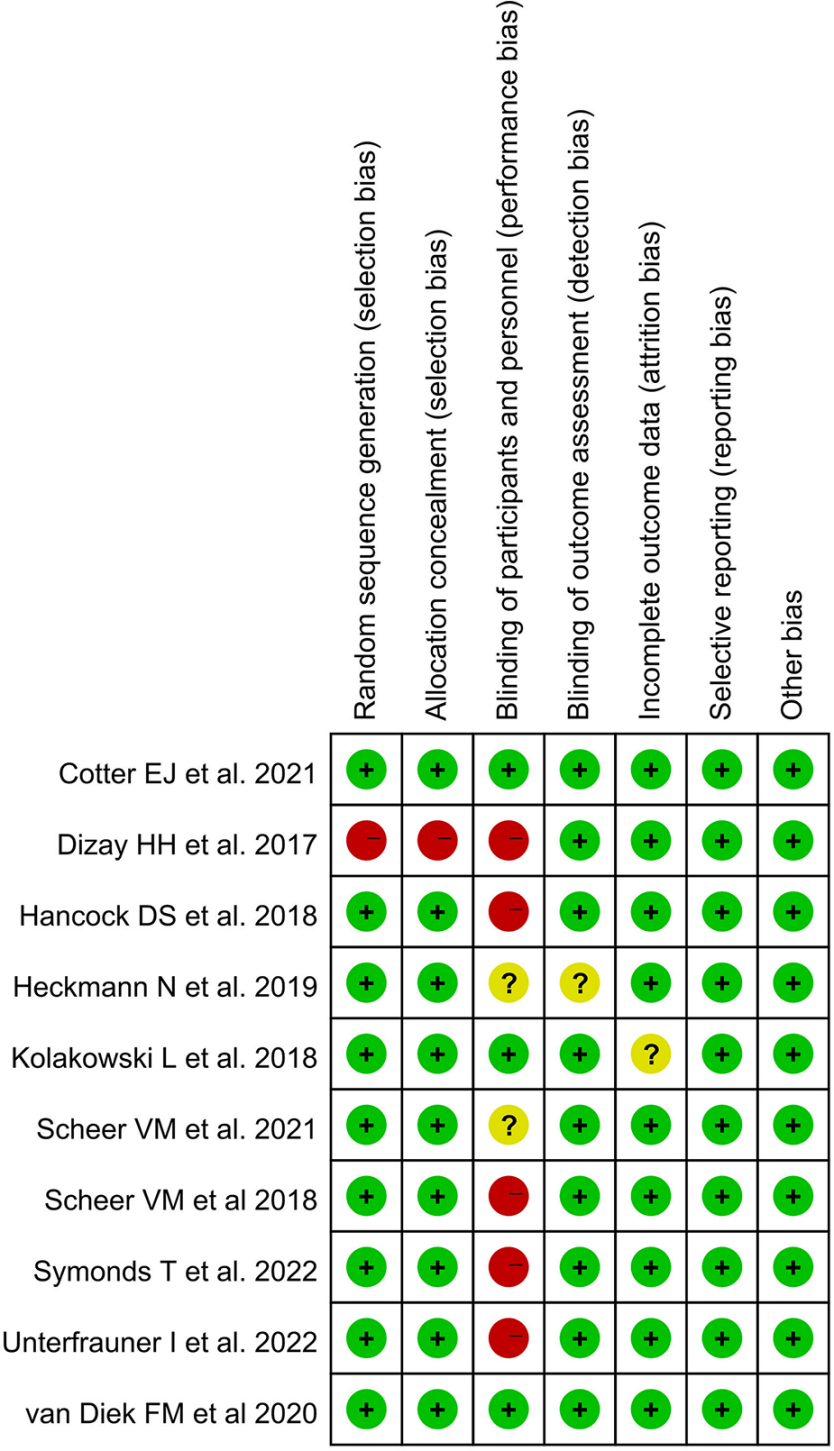
Risk of bias summary.

**Figure 3:**
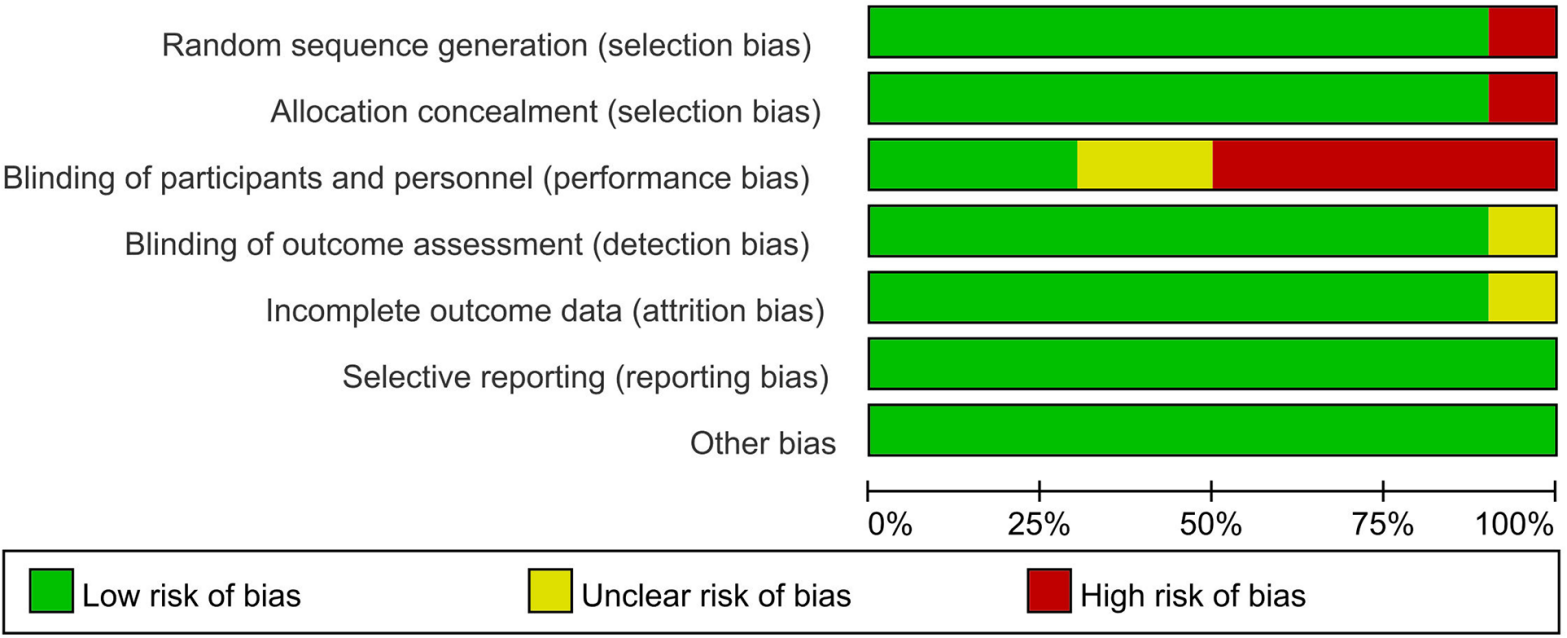
Risk of bias graph.

Only one study (34) was at high risk of selection bias because participants compared themselves before and after using BPO. Three studies (26, 33, 37) were at low risk of performance bias, and one study (36) was at unclear risk of detection bias. In addition, only one study had an unclear risk of attrition bias that may have obscured the analysis results (33). All studies were low risk in selective reporting and other biases. The Kappa score was 0.82 among reviewers (Figures 2, 3).

### 3.3. The rate of positivity for *Cutibacterium acnes* in different 5% BPO groups vs. non-BPO groups

A total of 9 articles were included in this analysis (25–28, 32, 34–37). In the 5% BPO vs. non-BPO subgroup, the results indicated that the rate of positivity for *C. acnes* was significantly lower in the 5% BPO group [OR, 0.16 (0.09, 0.27), *I*^2^ = 5, *p* < 0.00001].

In 5% BPO + clindamycin vs. non-BPO, the results indicated that the rate of positivity for *C. acnes* was significantly lower in the 5% BPO with clindamycin group [OR, 0.20 (0.11, 0.40), *I*^2^ = 0, *p* < 0.00001].

**Figure 4:**
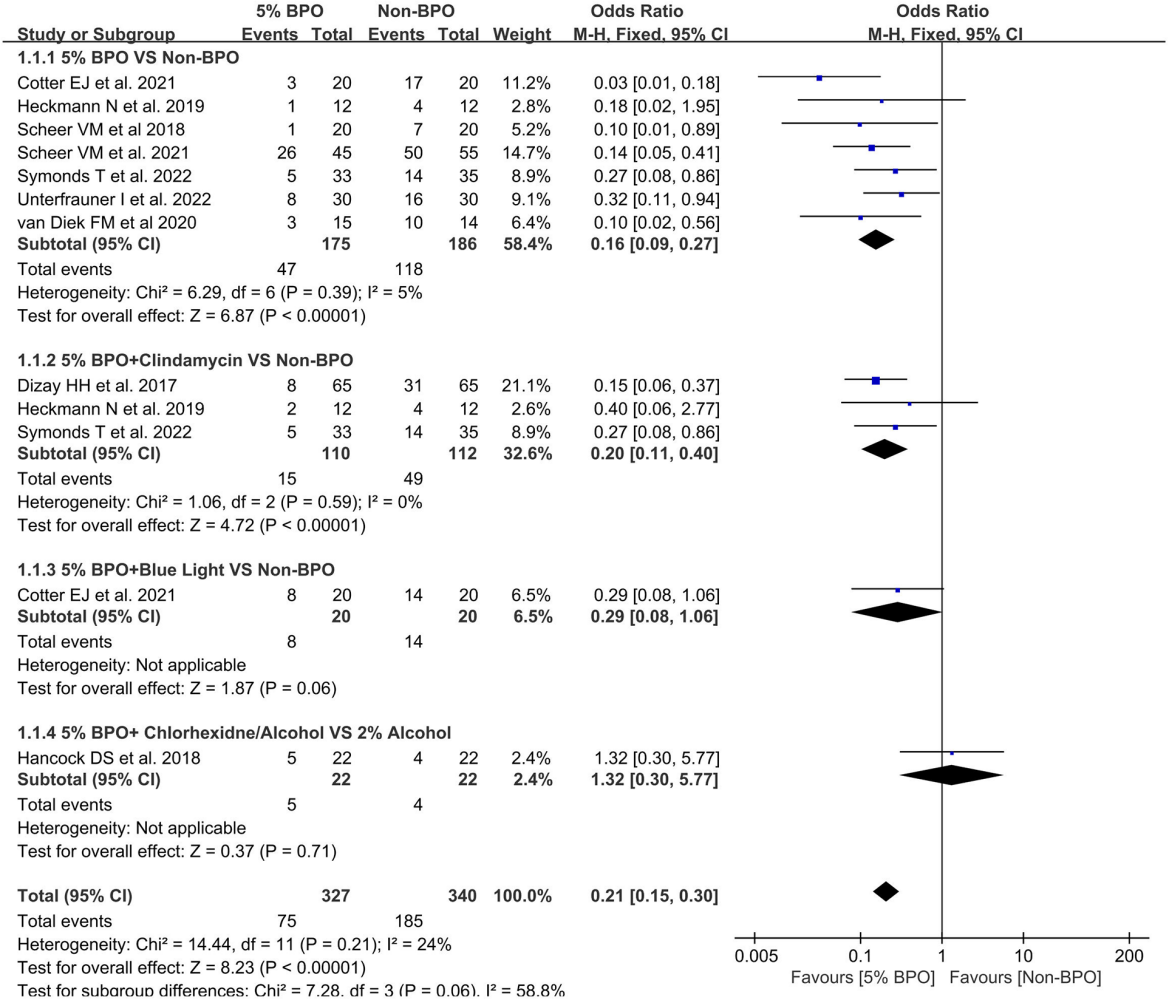
Forest plot showing the rate of positivity for Cutibacterium acnes in different 5% benzoyl peroxide groups vs. non- benzoyl peroxide groups.

Collectively, the 5% BPO group had a lower risk of *C. acnes* positivity [OR, 0.21 (0.15, 0.30), *I*^2^ = 24, *p* < 0.00001]. The test for subgroup differences was 58.8% (Figure 4).

### 3.4. The rate of positivity for *Cutibacterium acnes* with 5% BPO vs. 5% BPO with clindamycin

**Figure 5:**
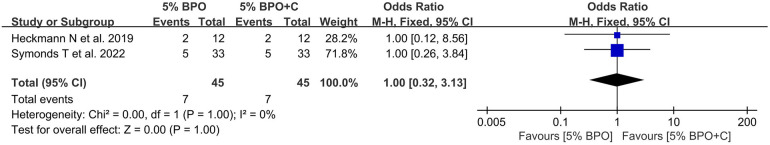
Forest plot showing the rate of positivity for Cutibacterium acnes with 5% benzoyl peroxide vs. 5% benzoyl peroxide with clindamycin.

A total of 9 articles were included in this analysis (25, 26, 32–34, 36, 37). The pooled result found no significant difference between the BPO group and the BPO with clindamycin group [OR, 1.00 (0.32, 3.13), *I*^2^ = 0, *p* = 1.00] (Figure 5).

### 3.5. Adverse events

**Figure 6:**
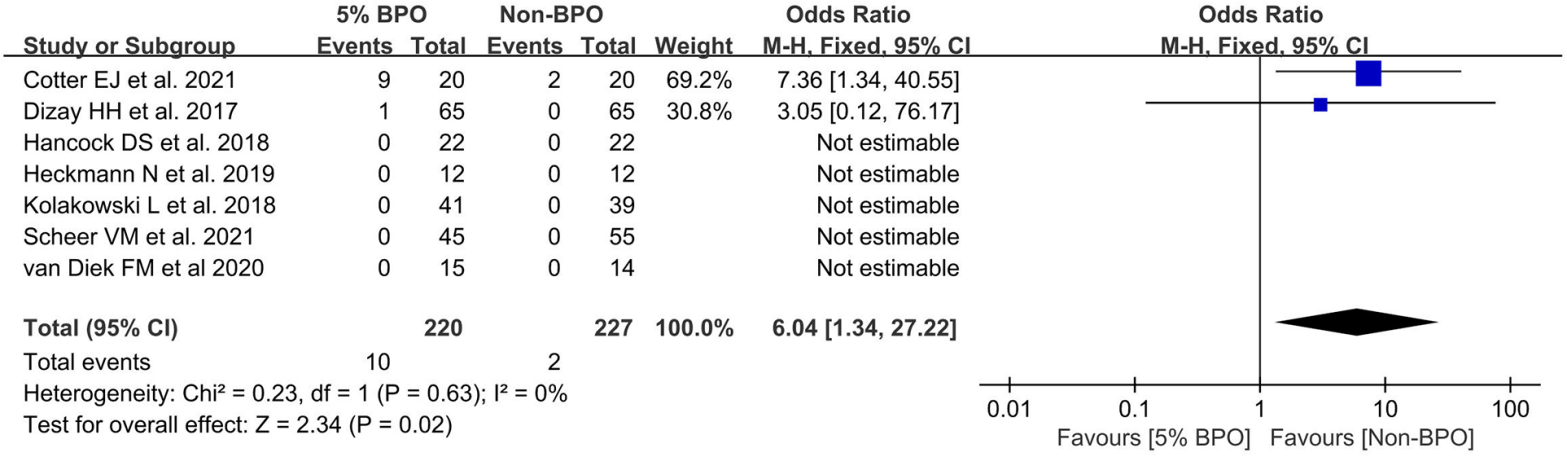
Forest plot showing adverse events.

A total of 7 articles reported adverse events (25, 26, 32–34, 36, 37). The results indicated that the rate of lower adverse events was significantly higher in the non-5% BPO group [OR, 6.04 (1.34, 27.22), *I*^2^ = 0, *p* = 0.02] (Figure 6).

### 3.6. Publication bias

**Figure 7:**
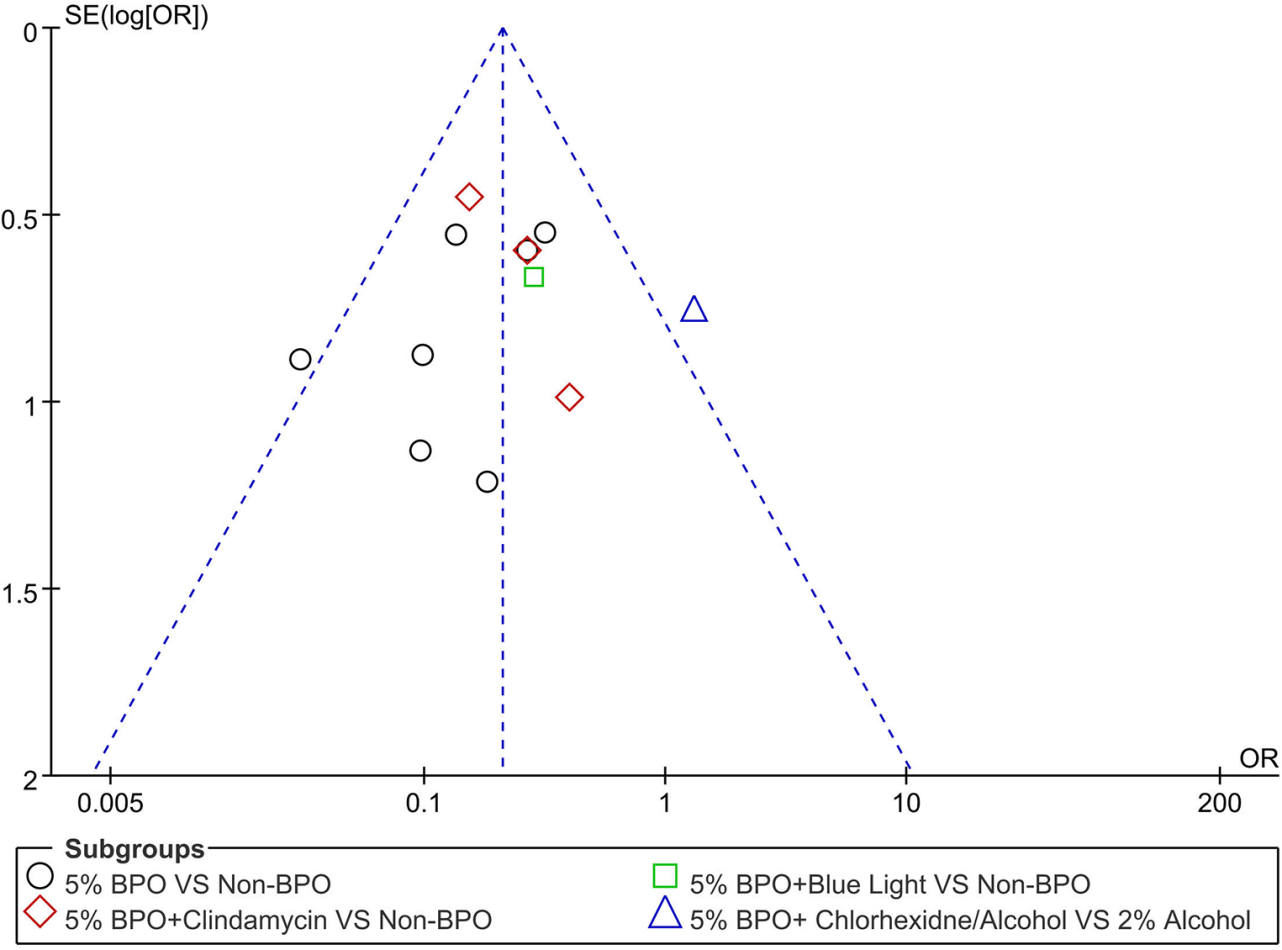
A funnel plot showing the positive C. acnes rate in different 5% benzoyl peroxide groups vs. non- benzoyl peroxide.

A funnel plot of the positive *C. acnes* rate in different 5% BPO groups vs. non-BPO was performed to ensure that this study had publication bias. This funnel plot summarized a mild degree of asymmetry, indicating a publication bias in the data (Figure 7).

## 4. Discussion

In this meta-analysis, we found that BPO significantly reduced the amount of *C. acnes* in the skin, which is consistent with previous studies (23, 24). Theoretically, BPO and clindamycin (antibacterial) are synergistic in their activity (38–40). To better explore the method of BPO, we conducted a comparative analysis of BPO alone or combined with clindamycin, but the results showed no significant difference. This pooled result was similar to those of a double-blind clinical trial that found no significant difference in efficacy between the combination therapy of niosomal BPO 1% and clindamycin 1% compared with niosomal clindamycin in acne vulgaris (41). Therefore, the combined use of clindamycin is unnecessary based on the current results.

As a drug to inhibit the proliferation of *C. acnes*, BPO is often used in the surgical area before surgery. In the literature we included, the use of BPO is not uniform. However, it is worth noting that BPO is used more than 3 times in most studies. This is because a single use of BPO is not effective in preventing infection (32, 34). Conversely, the longer the application time is and the more applications there are, the stronger the effect of BPO. Dizay et al. found that when BPO was applied only once, the gel is two-thirds effective in eliminating surface colonization. When used more than once, the efficiency will reach approximately 80%. Therefore, BPO should be used several times to achieve good results.

In addition to the number of BPO applications, gender also affected the outcome. *C. acnes* prefers to live within pilosebaceous glands (42). In the literature we included, two of the patients were male, and in the other several pieces of literature, the proportion of males was much higher than that of females. Chuang et al. found that despite standard skin preparation and prophylactic antibiotics, men still have higher colonization rates than women (43). In the use of BPO to prevent infection, if the ratio of men to women included in the two groups is inconsistent, the authenticity of the results may be masked. At the same time, it is also worth considering whether excluding all male patients would underestimate the effect of BPO.

At present, the indications for the application of BPO are mostly concentrated in dermatology, and the indications for the use of BPO to prevent infection in the shoulder joint have not yet formed a unified understanding due to the lack of literature. However, there has been much literature on risk factors for shoulder infections, including age, sex, hair, history of surgery, cortisone injections prior to surgery, and diabetes (44–47). Therefore, BPO can be used to reduce the likelihood of shoulder infection in patients with multiple risk factors.

The adverse events of topical BPO include mild dryness, concentration-dependent irritant dermatitis with erythema, angioedema, scaling and itching (48–50). In this study, we also found a similar situation. Direct contact with the epidermal active ingredient is the basis for BPO side effects (50). Once symptoms are detected, benzoyl peroxide use should be discontinued (51).

BPO is an over-the-counter, FDA-approved prescription medication that has long been used in dermatology (39). It breaks down into benzoic acid and hydrogen peroxide in the pilosebaceous duct, releasing free radicals against *C. acnes* (52). Compared with other antibiotics for surgical infection prevention, there was no resistance to benzoyl peroxide (53). BPO is usually formulated at concentrations of 2.5%, 5%, and 10%. However, the optimal use of BPO for shoulder prophylaxis has not been determined. In a network meta-analysis study, the combinations of BPO with adapalene or clindamycin (54% vs. 35% or 49% vs. 35%) were a more effective treatment for acne than BPO alone (54). Whether there is a similar efficiency in preventing shoulder infection remains to be further verified.

In addition, when using BPO to prevent shoulder infection, it is also necessary to pay attention to patients’ skin allergies to BPO. A patient who had undergone total knee arthroplasty developed a systemic reaction and intractable pain. A patient undergoing total knee replacement developed systemic reactions and intractable pain due to hypersensitivity to BPO (55). Bircher A et al. reported five patients who had relevant sensitization to BPO with complications from a knee or a shoulder joint implant (56). Typical symptoms include pain, swelling, and inflammation of the skin.

From the payer's perspective, BPO is a cost-effective strategy for preventing infection. A randomized controlled study reported that the application of BPO three times cost less than $10 per patient (33). In contrast, the minimum institutional cost of treating arthroscopic rotator cuff repair infection was $24,991.31 (57). The average total cost of a shoulder prosthesis infection was $46,745 (58). In addition, a recent study estimated that joint infections around hip and knee implants will cost $1.85 billion a year in hospital costs by 2030 due to increased surgery volumes (59). Two break-even analysis studies suggested that using BPO for infection prevention in shoulder surgery is a highly economically justified practice (57, 58).

## 5. Limitations

This study has several limitations. Most importantly, the nine studies obtained from the same journal may have caused bias in the results. Second, some studies involved participants without shoulder surgery, which could have influenced the results. Third, more studies are needed to analyse the effect of BPO on the specific colony number of *C. acnes*. At the same time, more literature is needed to compare the effect of BPO at different concentrations (2.5%, 5%, 10%) on the prevention of surgical infection of the shoulder joint.

## 6. Conclusion

BPO can decrease *C. acnes* in the shoulder to prevent infection. However, the combination of BPO and clindamycin does not enhance this effect further.

## Data Availability

The original contributions presented in the study are included in the article/Supplementary Material, further inquiries can be directed to the corresponding author.
